# Tomography and Prognostic Indices in the State of the Art of Evaluation in Hospitalized Patients with COVID-19 Pneumonia

**DOI:** 10.3390/pathogens11111281

**Published:** 2022-11-01

**Authors:** Sergio Críales-Vera, Huitzilihuitl Saucedo-Orozco, Pedro Iturralde-Torres, Gustavo Martínez-Mota, Estefanía Dávila-Medina, Verónica Guarner-Lans, Linaloe Manzano-Pech, Israel Pérez-Torres, María Elena Soto

**Affiliations:** 1 Department of Computed Tomography, Instituto Nacional de Cardiología Ignacio Chávez, Juan Badiano 1, Col. Sección XVI, Del. Tlalpan, Mexico City 14080, Mexico; 2 Cardioneumology Department, Instituto Nacional de Cardiología Ignacio Chávez, Juan Badiano 1, Col. Sección XVI, Del. Tlalpan, Mexico City 14080, Mexico; 3 Cardioneumology Department, Specialty Hospital, National Medical Center “La Raza” Mexican Social Security Institute, Seris and Zachila, La Raza, Azcapotzalco, Mexico City 02990, Mexico; 4 Instituto Nacional de Cardiología Ignacio Chávez, Juan Badiano 1, Col. Sección XVI, Del. Tlalpan, Mexico City 14080, Mexico; 5 Department of Physiology, Instituto Nacional de Cardiología Ignacio Chávez, Juan Badiano 1, Col. Sección XVI, Del. Tlalpan, Mexico City 14080, Mexico; 6 Department of Cardiovascular Biomedicine, Instituto Nacional de Cardiologíaa Ignacio Chávez, Juan Badiano 1, Col. Sección XVI, Del. Tlalpan, Mexico City 14080, Mexico; 7 Department of Immunology, Instituto Nacional de Cardiología Ignacio Chávez, Juan Badiano 1, Col. Sección XVI, Del. Tlalpan, Mexico City 14080, Mexico; 8 Line of Cardiovascular Research of the American British Cowdray Centro Medico ABC IAP, México Sur 136 116, Las Americas, Álvaro Obregón 01120, Mexico

**Keywords:** chest tomography, combined indexes, COVID-19, classification of severity

## Abstract

Evaluation in medical emergencies of COVID-19 patients represents a challenge to regulate preventive and timely management. There are key imaging and laboratory tools to classify the severity. The aim of the study was to evaluate the chest CT score performance and prognostic indices in COVID-19 patients to predict the progression to critical illness. This was a retrospective study between run between April and December 2020, in which 109 patients were included. Patients of any age and gender and who required hospitalization due to a confirmed COVID-19 diagnosis by RT-PCR and chest CT and laboratory were analyzed. In 75% of them, there was at least one comorbidity, and 30% developed critical illness, and the average mortality was 10%. In 49.5%, there was a CORADS-5 on admission, and in 50%, there was a peripheral distribution of the interstitial infiltrate in the left lower lobe. The risk factors were FiO_2_, CT score > 18, and the NRL index. The combination of the high-risk Quick COVID-19 Severity Index (qCSI) plus CT score > 18 indices was the best prediction index for the development of a critical condition. The combined use of indices in infected COVID-19 patients showed diagnostic accuracy and predicted severity. Imaging and the laboratory tests are key tools independent of the wave of recurrence.

## 1. Introduction

Chest-computed tomography (chest CT) has shown great utility in the diagnosis of infection with SARS-CoV-2. Its complementary use with the reverse transcription polymerase chain reaction (RT-PCR) test to assess subjects infected with SARS-CoV-2 allows timely medical intervention and accurate therapeutic decision in COVID-19 [1].

The most common COVID-19 findings in chest CT are ground glass opacities (GGO), which are isolated or in combination with areas of focal consolidation. These areas show a predominantly bilateral distribution and sub-pleural predominance [2,3,4]. In patients with SARS-CoV-2 infection, chest CT provides relevant data that are currently part of the diagnostic tools [5,6] and might be further implied in prognosis [7,8].

In March 2020, the Dutch Society of Radiology developed a standardized evaluation scheme for the lung condition due to COVID-19, the acronym of which is CO-RADS. This system proposed a level of suspicion of pulmonary involvement in COVID-19 [9]. It is also possible to evaluate the severity of lung damage and extension caused by COVID-19 through chest CT in a semi-quantitative way [10], and there is an adequate correlation between the severity and the degree of affection [11]. One of these methods is the CT score, which is an index that has shown an adequate correlation with age and inflammation. A CT score value ≥ 18 predicts short-term mortality in the follow-up of patients with COVID-19 [12].

The use of predictive clinical indexes to facilitate medical judgment, leads to an improvement in the therapeutic and clinical decisions determining whether the patient with SARS-CoV-2 infection can be cared for at home or requires hospitalization. These indices are easy to obtain and are useful in risk stratification of complications, respiratory deterioration, and prognostic determination of mortality as the CURB-65 in community-acquired pneumonia [13,14]. COVID-GRAM helps to evaluate the risk of critical illness in hospitalized patients, and it predicts the risk to be admitted to the critical care unit (ICU), the requirement of invasive ventilation and of death [8]. Similarly, the Quick COVID-19 Severity Index (qCSI) predicts the risk of critical respiratory disease in 24 h [15].

On the other hand, another index, the neutrophil–lymphocyte ratio (NLR), predicts the level of physiological stress [16]. It allows for the early detection of sepsis and helps in decision making regarding the requirement for admission to the ICU [17]. Therefore, many of these indexes allow a comprehensive evaluation of the underlying problem in the patient at the time they seek care. Throughout the epidemic, there has been viral variation, and pneumonia in patients infected with SARS-CoV-2 also occurs in post-vaccinated individuals. Survival improves with early detection and cases where imaging and laboratory tools remain state-of-the-art [18] and have shown less lung involvement and clinical severity. The objective of this work was to evaluate the prognostic performance of clinical indexes and chest CT in patients with COVID-19 who progress to critical illness.

## 2. Materials and Methods

This was a retrospective and observational study run between April and December 2020. Patients of any age with a confirmed diagnosis of COVID-19 by RT-PCR [19,20,21,22] had undergone chest CT on admission and received standard treatment according to the recommendations available at the date of admission by the American Infectious Diseases Society (IDSA), and the panel of the National Institutes of Health (NIH) was included [23,24]. Patients with a diagnosis of acute coronary ischemic syndrome (ACS), acute heart failure, patients who were admitted to another clinical trial, those who developed critical illness in the first 24 h after admission, or those who did not have a RT-PCR result were excluded. We conducted the present study following the Helsinki declaration. The protocol was accepted by the INC Ethics and Research Committee (research protocol number 17-1033) and registered at www.clinicaltrials.gov (NCT04557345), accessed on 1 January 2020. All patients signed informed consent before the start of the investigation.

Critical illness was defined as the need for supplemental oxygen (>10 L /min by low-flow device, high-flow device, non-invasive, or invasive ventilation) or death during patient hospitalization [8,25]. Data from the electronic medical record at admission to the emergency service, a clinical questioning, and a physical examination were collected. Oxygen saturation was quantified [26,27].

### 2.1. Model Development

A univariate analysis of the clinical variables and of the laboratory and chest CT characteristics was performed. The diagnosis of COVID-19 was made according to the interim guidelines of the World Health Organization (WHO) 2019 [28] and the diagnosis and treatment guidelines of COVID-19 in China [29].

Non-serious patients met early-onset epidemiological characteristics [30], epidemiological history [31], fever or other respiratory symptoms [32], and typical abnormalities of the CT scan of the pneumonia virus [33]. Critically ill patients also met at least one of the following conditions: shortness of breath, respiratory rate ≥30 times/min, oxygen saturation (resting state) ≤93% [3], PaO_2_/FiO_2_ ≤ 300 mmHg. COVID-19 patients were confirmed by a positive high-throughput sequencing or positive RT-PCR result for SARS-CoV-2 RNA from nasal and throat swab samples [1].

Anthropometric and demographic characteristics, and a history of comorbidities such as systemic arterial hypertension [34], type 2 diabetes mellitus [35], dyslipidemia [36], chronic renal failure [37], and arterial blood gas were examined [38]. The levels of laboratories were obtained at the moment of admission.

Prognostic scores and indexes were calculated. Web-based risk calculators were used: for COVID-GRAM (available at http://118.126.104.170/ [8], accessed on 1 January 2020), The National Early Warming Score 2 (NEWS2) [39] and qCSI (available at https://covidseverityindex.org/ [15], accessed on 1 January 2020). The neutrophil–lymphocyte ratio was calculated with the following formula: NLR = (absolute neutrophil count, cells/(absolute lymphocyte count) [16].

### 2.2. Computed Tomography

Images were acquired with a Siemens 256-slice multidetector tomograph (Somatom^®^ Definition Flash 128 × 2, Siemens Healthcare, Forchheim, Germany). The chest scout was acquired using 35 mA, 100 Kv, and 6 mm slices, and with the chest tomographic slices maintaining inspiration in a cephalocaudal direction with 80 mA, 100 Kv, a duration of 2.24 s, a pitch of 1, and slices of 1 mm, with a total of 110 DLP, with which a total of 1.5 mSv is calculated with the conversion factor for the thorax. Multiplanar reconstructions were performed with Kernel filters B26f and B50f for the mediastinum and lung, respectively, at 1 mm slices.

The CT score was calculated by 2 experts as follows: a value of 0 to 5 was assigned in each lung lobe for a total of five lobes (assigning 0 points with involvement was 0%, 1 point with involvement less than <5%, 2 points with involvement of 5–25%, 3 points with involvement of 26–50%, 4 points with involvement of 51–75%, and 5 points with involvement of >75%), obtaining a severity score by tomography of 0–25 points [11]. There was a strong agreement between raters (0.89).

### 2.3. Statistical Analysis

Variables were expressed in frequencies and percentages, mean ± SD, mean, or interquartile range. The comparisons of proportions between groups were performed by the chi-squared test or Fisher’s exact test and Student’s T test or Mann–Whitney U. A logistic regression analysis was performed [40]. A multivariate model was used in the univariate analysis to select the predictors. We report sensitivity, specificity, and the positive and negative predictive value. Statistical analysis was performed with STATA version 16.0 software (Stata Corp LLC, Los Angeles, CA, USA).

## 3. Results

Eighty-six patients were excluded due to a lack of clinical data, or delayed RT-PCR or chest CT results, and three patients were also excluded who required endotracheal intubation within 24 h of admission. Therefore, 109 patients were included. Table 1 shows the characteristics and demographics of the included patients. Critically ill patients had been admitted with respiratory rate breaths/min well above normal, and a greater age and a higher percentage of cerebrovascular events; this had a significant statistical difference versus those who did not have critical illness.

Table 2 shows the laboratory findings and the differences observed according to the severity of the disease. The parameters of respiratory compromise showed a lower PaO_2_, mmHg, Horowitz Index (P/F ratio), and a lower FiO_2_; the (%) increased, and this difference was statistically significant in patients that evolved to critical illness. The neutrophil cell count, x103/L, and the lymphocyte-to-neutrophil ratio were found to be increased in critically ill patients. In this group of patients, the ranges of glucose, creatinine, blood urea nitrogen, eGFR, C-reactive protein, D-dimer level, hsTnl, NT-proBNP, creatine kinase, CK-MB fraction, ferritin, fibrinogen, alkaline phosphatase, and LDH were increased. The classification of patients according to indexes is shown in Table 3. Critically ill patients had a CURB score of >2 versus non-critically ill patients, and this was statistically significant. The risk of mortality was greater than this score, being 6.8, which is a recommended for short hospitalization and close surveillance. The mortality found was related to the increase in the CURB score in critically ill patients. The COVID-GRAM index showed that a score range considered for intermediate risk is more frequent in non-critically ill patients. This index falls into the high-risk score more frequently in critically ill patients having a statistical significance. The News 2 index, which determines the degree of illness of a patient, was indicated to request an intervention in critical care for the patient, and it showed that there was a higher frequency of patients with low risk in the non-critical versus the critically ill. The high risk was higher in the latter who evolved to critical illness. The same differences were observed in the Berlin index to define respiratory distress syndrome, and the COVID-19 rapid severity index (qCSI) that predicts the risk of critical respiratory illness within 24 h in patients admitted from the emergency department. We show an obvious statistical difference to separate critically ill patients with COVID-19.

Table 4 shows the tomography findings according to the severity of the patients. GGO, CORADS-5, and the peripheral distribution of the interstitial infiltrate are shown. In critically ill patients evaluated by CT findings, pleural and pericardial effusion defines statistical differences between seriously ill and non-critically ill patients. CORADS in this series did not show differences between these clinical states, but these differences were demonstrated by the TSS of severity.

Table 5 shows in the multivariate analysis of the variables that impact on the prognosis, and Table 6 describes the determination of the sensitivity, specificity, and predictive values for score by CT in critical illness.

**Figure 1:**
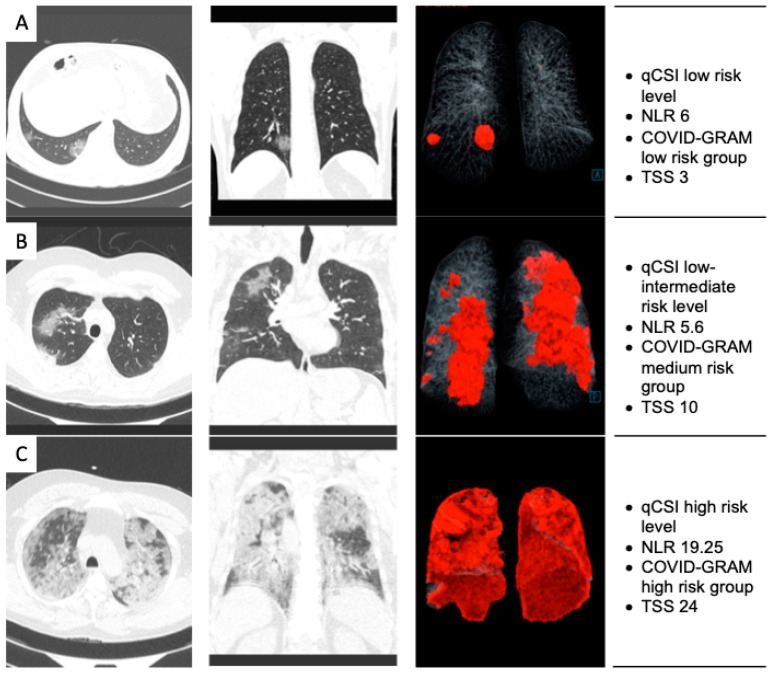
(**A**) Image of computed tomography in lung window, axial, and coronal section, as well as representation by artificial intelligence with quantitative analysis. Ground glass areas are demonstrated in the posterior and lateral segments of the right lower lobe. CT Score 3/40. (**B**) Image of computed tomography in the lung window, axial, and coronal section, as well as representation by artificial intelligence with quantitative analysis, showing areas of ground glass in the upper right lobe and other scattered areas in the left lung. CT Score 10/40. (**C**) Image of computed tomography in lung window, axial, and coronal section, as well as representation by artificial intelligence with quantitative analysis, showing areas of ground glass with diffuse distribution in both lungs. CT Score 24/40.

Figure 1 shows computed tomography images of patients in different severity conditions. A total of 19/109 (17%) died. The results of the development of the model in Table 5 shows univariate and multivariate analysis.

Table 6 shows the sensitivity, specificity, positive predictive value, negative predictive value, positive likelihood ratio (LR+), negative likelihood ratio (LR-), and OR of each predictor in simple and combined form.

**Figure 2:**
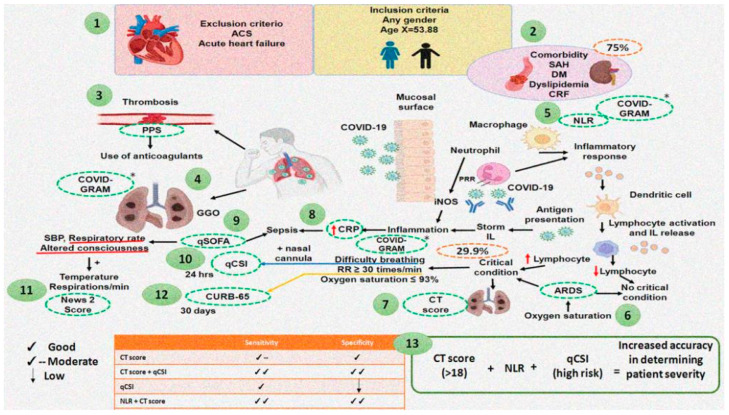
Summary of how we consider the evaluation in the study. 1. Inclusion and exclusion criteria of the study. 2. Example of comorbidities of study patients. 3. Use of Padua Prediction Score for Risk of VTE. 4. The most common COVID-19 findings in Chest CT are ground glass opacities, also used in the COVID-GRAM. 5. Development of neutrophil–lymphocyte ratio. 6. The acute respiratory distress syndrome allows classifying patients in critical and non-critical condition. 7. The CT score, with which we could predict which patient would require priority ventilator support and hospitalization. 8. The CRP is used to discriminate probable bacterial from non-bacterial infections, and also to assess the severity of the disease. 9. qSOFA is used for the prediction of mortality; however, these are more frequently used for the diagnosis of sepsis, which occurs in critically ill COVID patients. 10. The qCSI Predicts 24-hr risk of critical respiratory illness in patients admitted from emergency department with COVID-19. 11. Respiratory rate, altered of consciousness, temperature, and respirations/min comprise News 2 score. 12. The CURB-65 Score for Pneumonia Severity. It is a mortality prediction scale used in patients with community-acquired pneumonia. 13. With the combined use of indices such as the CT score, neutrophil–lymphocyte ratio, Quick COVID-19 Severity Index, we can classify and select with greater precision the severity of the patient infected by SARS-CoV-2. Abbreviations: ACS = acute coronary ischemic syndrome, CRF = chronic renal failure, CRP = C-reactive protein, CT = computed tomography, DM = diabetes mellitus, GGO = ground glass opacities, NLR = neutrophil–lymphocyte Ratio, PP = Padua Prediction Score, Qcsi = Quick COVID-19 Severity index, qSOFA = quick SOFA, SAH = systemic arterial hypertension.

The critically ill patients were older, the respiratory rate was higher, and the tomography showed a higher frequency of consolidation and pericardial effusion. The risk factors were increased FIO_2_% and increased neutrophil–lymphocyte ratio. The COVID-GRAM and qCSI score was seven times higher in critically ill patients; Figure 1 and Figure 2 show and summarize how we consider the evaluation in the study.

## 4. Discussion

During the COVID-19 pandemic, the provision of optimal treatment for patients emerged as a priority, which led to the implementation of measures with multiple therapies. At the same time, protecting health workers involved in the management and care of these patients also became a priority [40]. Additionally, several clinical indexes were proposed by many creators aiming at the evaluation and selection of the patients that might receive therapy at home, those that required hospitalization, and those that needed ventilatory support. It is unknown whether the new variants will have the same degree of pulmonary involvement and functional impact, and, therefore, it is necessary to continue the clinical and tomographic evaluation in patients with COVID-19 infection.

Many indexes were used during the pandemic, including the CURB-65 Score for Pneumonia Severity [41].

We found that the probability of mortality predicted by CURB 65 was increased in our series as in other studies. A comparison between the indices proposed to predict mortality and to determine the need for invasive mechanical ventilation showed that in regard to the accuracy between the CURB-65 Score, Pneumonia Severity Score (PSI), and COVID-GRAM in patients with COVID-19, the COVID-GRAM index was more accurate in identifying patients with higher mortality from SARS-CoV-2 pneumonia [13]. None of these scores accurately predicted the need for invasive mechanical ventilation on admission to an intensive care unit [42]. In China, a score (COVID-GRAM) was developed for hospitalized patients and their risk of critical illness. The authors did not inform about the indications for admission. Among the parameters determined by the COVID-GRAM is the anomaly of the chest radiographic study, as well as the presence of dyspnea, and the most important thing is that it includes the NLR index among its parameters. In our findings, this last biomarker is the one that seems to be decisive and is a simple and accessible laboratory determination that predicts well the concept of severity in these patients when added to the contribution of image data.

This score COVID-GRAM had internal validity in China; however, the external validity needed to be evaluated in other series. In this study, we found that patients classified as low risk did not die; however, there was a percentage of 13% and 30% in patients with intermediate and high risk, respectively, which allowed us to determine that the COVID GRAM index classifies patients well into patients who present a risk of being critically ill due to COVID-19, and with another utility to predict mortality. However, we consider that the visual change tool provided by the tomography image makes the changes very specific, and these can go hand in hand with the changes in inflammation which can be provided by common laboratory tests easy to apply and measure in serious circumstances.

In addition to the evaluation by these scores, other tools emerged at the same time to determine patients with a specific need for ventilatory support [43].

On the other hand, it is necessary to know if intubation is necessary after a high-flow nasal cannula (HFNC), and this risk predicts the failure of the HFNC and the need for intubation after its use during the care of a hospitalized patient. The ROX index was suggested in this evaluation; however, we did not find this in the present study.

Other criteria to predict the percentage of mortality were the 4C Mortality Score for COVID-19 and inpatient risk [44]. In this study, we found that this score predicts mortality in a similar way to that proposed by the author of the score, and it classifies the probability of mortality in hospitalized patients.

We were also unable to assess the score of the Brescia-COVID Severity Scale/Algorithm. This was created in April 2020 with a unique approach to the step-by-step management of patients with COVID-19 based on clinical severity [45]. We found it complicated to collect the data in this scale retrospectively. The need to decide whether or not to give anticoagulation to hospitalized patients with this risk or the need to define the contraindication due to the risk of bleeding was raised, for which some criteria were proposed to evaluate these conditions. The Padua Prediction Score for Risk of VTE criteria to determine the need for anticoagulation in patients hospitalized due to risk of VTE has also been suggested, and, in this series, a total of 98 individuals had high risk, and among them, 19 (19%) died. However, they include heart and respiratory failure within their scoring parameters [46]. In all of them, the authors found usefulness, and suggested their employment during the classification and follow-up of patients with COVID-19. Some other indexes, such as the qSOFA (Quick SOFA) Score for sepsis, were also proposed for the prediction of mortality; however, these are more frequently used for the diagnosis of sepsis, which occurs in critically ill COVID patients.

Among the indices that evaluate respiratory compromise is the NEWS. This score was analyzed in 35,585 subjects, including respiratory rate, oxygen saturation, supplemental oxygen, temperature, and systolic blood pressure to classify the degree of disease and the type of intervention required in intensive care. The authors suggested confirming the reliability of their scales [39], and in NEWS 2, the score determines the degree of illness of a patient to request intervention in critical care, and determines the urgent review of the doctor, so that they can decide if they need to intensify the car. We saw that in patients with a low or intermediate score, the prevalence of mortality was 0, and in those classified as high risk, the mortality was high. Therefore, we consider that this index defines when the patient requires an optimal escalation in attention and confirms the author’s findings. However, there are other indices for critical illness in COVID-19 [47].

The Quick COVID-19 Severity Index (qCSI), which predicts the risk of critical respiratory illness in 24 h in patients admitted to the emergency department with COVID-19, showed that the score discriminated well between subjects who had a low and high risk, but there is a percentage of individuals with intermediate risk who were likely to have critical respiratory decompensation of the disease and death. We, therefore, believe that at least 20% of our series had this risk, and we did not include it in intermediate scores to all individuals with higher risk. The Berlin study for the diagnosis of ARDS performed better on our data in predicting ARDS [48].

Several studies have evaluated the usefulness of nonspecific biomarkers, such as C-reactive protein, white blood cell count (WCC), and absolute neutrophil count (ANC) [49,50]. We found them useful, as they accurately distinguished patients with high and low risk of acute respiratory distress syndrome (ARDS), and this correlates with the time when subjects were classified as high risk and had a higher percentage of mortality.

Chest CT, an imaging parameter, is useful in the evaluation of patients with lung damage or severe complications of viral pneumonia, mainly when the chest X-ray finding is normal or inconclusive [51]. In this study, we found that CT provides a rapid assessment and predicts critical severity in the short term [4,11]. Using the CT score, we were able to predict which patient would require priority ventilator support and hospitalization. The use of the indices, alone or combined, even when evaluated retrospectively, allowed us to recognize which parameters make the difference to define between a state of health without severity and with a high probability of being in severity.

We found the use of CT as the only diagnostic test that had good specificity and moderate sensitivity; however, when its use is combined with other indices, such as qCSI, these increases, and the VPP and NPV also increase. In a study by Liang et al. [8], the authors sought the validation of a clinical score to predict the occurrence of critical illness, and found that the associated factors were chest X-ray abnormalities, hemoptysis, dyspnea, state of unconsciousness, number of comorbidities, previous cancer, NLR index, lactic dehydrogenase, and direct bilirubin, and the area under the curve was 0.88; they concluded that the scale predicts patients who will develop critical illness. In our study of the proposed parameters, we also showed statistical robustness when comparing critical and non-critical patients.

In this study, we analyzed the tomographic status together with fundamental laboratory parameters to evaluate patients with COVID-19 at the time of admission, as was suggested by Li X et al. The usefulness of computed tomography for the diagnosis and prediction of mortality from COVID-19 was evaluated, and we obtained similar findings [52].

The Quick COVID-19 Severity Index (qCSI) was included for the evaluation of its three-variable scale, respiratory rate, pulse oximetry, and nasal cannula flow. Respiratory rate is a parameter that is included in the scores of other indices and appears to be of great importance. The qCSI index was widely used by some countries during the pandemic. Studies comment that its results surpassed other models, including the evaluation of rapid sequential organ failure related to sepsis and that of the CURB-65. Therefore, this index was proposed as a useful clinical tool to help make decisions about the level of care required in patients admitted to a hospital [15].

In the present study, we found that the qCSI index showed good sensitivity and low specificity; however, its usefulness showed greater utility when combined with the CT evaluation, reaching a better specificity and a high percentage of NPV. This indicates that it predicts the probability for the individual to reach a severity condition when the score is not met. One of the accessible tools we have is the laboratory. In this pandemic, the RLN was proposed as a useful marker of an inflammatory state. In this study of critically ill patients with COVID-19, we found an area under the curve (AUC) of 0.65, with sensitivity and specificity of 0.37 and 0.90, respectively. Analysis of this index was performed in China and achieved an AUC of 0.84, sensitivity of 0.55, and specificity of 0.84. The explanations of the differences can be several, and one of them could be related to the day on which the index measurement was made.

The result would depend on whether the determination was made at the onset of symptoms, or when they were already in an advanced stage. It is a parameter that can integrate a systemic problem related not only to lung damage, but also to different organs. We evaluated the combined use of the LRN index with CT and found that sensitivity and specificity increased in relation to CT. The average CT Score in critically ill patients was 11.01. Of all the indexes used in critically ill patients, the combination of high-risk qCSI plus CT score > 18 was the most useful, emphasizing the importance of ventilatory care. The multivariate analysis showed that FiO_2_, CT Score > 18, and the NRL index are the main risk factors. This shows biological coherence, since the respiratory rate (breaths/min) was clearly increased, and the Horowitz Index (P/F ratio) decreased in critically ill patients.

On the other hand, the correlation between the CT Score and the respiratory rate (breaths/min) was moderate (40%) (*p* = 0.0001), which indicates that the parameters observed in the increase in respiratory work correlate with the damage found at the lung level reported by the CT Score. The use of clinical risk indices (COVID-19 and qCSI) in combination with a CT score > 18 obtained by CT of the chest are a good option in the evaluation and initial stratification of patients infected with SARS-CoV2, and they help to take accurate clinical decisions. 

Limitations. This was an observational, retrospective study. The admitted patients did not represent the behavior of mild cases of COVID-19 since our institution is a tertiary hospital. There was not the possibility of follow-up after discharge, and further evaluation was difficult. The FiO_2_ (%) provided to the patient fluctuated due to the method of administration. It was dynamic, and, therefore, the calculation had limitations. This could be improved if cases are evaluated post-infection and follow-up is suggested to the patient in future applications of this methodology.

## 5. Conclusions

The combined use of indices such as CT Score, neutrophil–lymphocyte ratio, and Quick COVID-19 Severity Index more accurately classify and select the severity of the patient infected by SARS-CoV-2. In Mexico, this allowed a better classification of patients with COVID-19 at the time of hospital admission and could guide towards optimal comprehensive therapeutic management in patients at risk of progressing to critical condition. Respiratory rate (breaths/min) is a clinical parameter that suggests severity in patients and is included in the vast majority of indices in their classification score. Therefore, it should be considered as a clinical parameter of impact, with relevant use for research beyond the time of any pandemic.

## Figures and Tables

**Table 1:** Clinical and demographic characteristics at hospital admission in patients diagnosed with COVID-19.

		Critical Illness		
Variable	Total (*n* = 109)	No (*n* = 77)	Yes (*n* = 32)	*p*
Age, years	53.88 ± 13.51 *	52.02 ± 13.21 *	57.90 ± 13.37 *	0.02 ^†^
Male gender (%)	69 (63.3)	46 (59.74)	23 (71.88)	0.23 ^‡^
Smoking (%)	22 (20.18)	18 (23.38)	4 (12.50)	0.19
**Admission** **Measurements (Range)**				
Symptom onset-hospitalization, days	6 (3–9)	6 (3–9)	7 (2–9.5)	0.94 ^§^
Respiratory rate, breaths/min	20 (18–26)	19 (18–22)	25 (22.5–32)	0.001
Heart rate, beats/min	93 (80–105)	90 (80–104)	95.5 (82.5–110)	0.17
Temperature, °C	36.6 (36–37.3)	36.5 (36–37.2)	37 (36.3–37.8)	0.08
MAP, mean (SD), mmhg	91.96 ± 13.24 *	92.27 ± 11.86 *	91.21 ± 16.28 *	0.70 ^†^
**Symptoms, Number and (%)**				
Fever	93 (85.32)	66 (85.71)	27 (84.38)	0.53 ^¶^
Shortness of breath	81 (74.31)	56 (72.73)	25 (78.12)	0.85 ^†^
Dry cough	93 (85.32)	64 (83.12)	29 (90.62)	0.24 ^¶^
Headache	53 (48.62)	38 (49.15)	15 (46.88)	0.81 ^†^
Sore throat	56 (51.38)	39 (50.65)	17 (53.12)	0.81
Myalgia/arthralgia	77 (70.64)	55 (71.43)	22 (68.75)	0.78
Diarrhea	20 (18.35)	14 (18.18)	6 (18.75)	0.94
**Comorbidities, Number and (%)**				
Number of Comorbidities				
0	35 (32.11)	27 (35.06)	8 (25)	0.30
1	34 (24.68)	19 (25.00)	15 (46.88)	0.02
2	23 (21.10)	18 (23.38)	5 (15.62)	0.02
3	9 (8.26)	9 (11.69)	0	0.03
4	5 (4.59)	3 (3.9)	2 (6.25)	0.46
5	3 (2.75)	1 (1.30)	2 (6.25)	0.20
Obesity	27 (24.77)	17 (22.08)	10 (31.25)	0.31 ^†^
Hypertension	52 (47.71)	37 (48.05)	15 (46.88)	0.91
Diabetes	31 (28.44)	20 (25.97)	11 (34.38)	0.37
Dyslipidemia	30 (27.52)	21 (27.27)	9 (28.12)	0.92
Cardiovascular disease	16 (14.68)	10 (12.99)	6 (18.75)	0.30 ^¶^
COPD	2 (1.83)	2 (2.60)	0	0.49
Cerebrovascular disease	3 (2.75)	0	3 (9.38)	0.02
Malignancy	1 (0.92)	1 (1.30)	0	0.70
Chronic kidney disease	10 (9.17)	8 (10.39)	2 (6.25)	0.39

Values are expressed in number (percentage), median (interquartile range). °C = degree Celsius, COPD = chronic obstructive pulmonary disease, MAP = mean arterial pressure, SD = standard deviation, * media (Standard deviation), ^†^ Student’s *t*-test, ^‡^ chi-squared test, ^§^ Mann–Whitney U test, ^¶^ Fisher’s exact test.

**Table 2:** Laboratory findings on admission in patients with COVID-19.

		Critical Illness		
Variable	Total (*n* = 109)	No (*n* = 77)	Yes (*n* = 32)	*p*
PaO_2_, mmHg	63.5 (51.5–80.5)	68 (55.5–85)	51.5 (46–64)	0.00 ^§^
FiO_2_, (%)	41 (29−50)	33 (21–41)	60 (41–60)	0.001
Horowitz Index (P/F ratio)	166.6 ± 97.08 *	221.95± 92.14 *	96.56 ± 47.21 *	0.001 ^†^
Hemoglobin, g/L (range)	14.6 (13.6–15.5)	14.6 (13.8–15.6)	14.5 (13.2–15.5)	0.68 ^§^
Hematocrit, (%)	44 (41–47.4)	44 (41.5–47.4)	44 (44–47.4)	0.98
Platelet count, ×10^3^/L (range)	221 (160–274)	211 (156–266)	238.5 (167.5–316)	0.14
Neutrophil cell count, ×10^3^/L	6.3 (4–9.7)	4.7 (3.3–8.0)	9.3 (6.2–13.6)	0.001
Lymphocyte count, ×10^3^/L	0.9 (0.6–1.3)	1 (0.7–1.4)	0.8 (0.5–1.0)	0.001
Neutrophil–lymphocyte ratio	6.3 (3.6–12.5)	5.12 (2.8–9.38)	11.04 (7.75–21.41)	0.001
Sodium, mmol/L (range)	135 (132.4–137)	135 (133–137)	134 (130.4–136.6)	0.13
Potassium, mmol/L (range)	4.11 (3.78–4.58)	4 (3.6–4.4)	4.38 (3.97–4.87)	0.02
Calcium, mg/dL (range)	8.3 (7.9–8.8)	8.37 (8.11–8.96)	8.2 (7.7–8.56)	0.01
Glucose, mg/dL (range)	119 (105.8–174.8)	113 (103–148)	136.4 (115.4–226)	0.001
Creatinine, mg/dL (range)	0.96 (0.75–1.3)	0.85 (0.72–1.1)	1.25 (0.8–1.9)	0.001
Blood urea nitrogen, mg/dL	17.8 (12.1–27.4)	16(11–20.6)	27 (19.2–46.7)	0.001
eGFR, mL/min/1.73 m^2^	82.3 ± 38.5 *	86.7 ± 38.21 *	57.37 ± 35.93 *	0.001 ^†^
C-reactive protein, mg/L	81 (27.5–192)	51.8 (17.2–118.6)	186 (99.5–278)	0.001 ^§^
D-dimer level, μg/mL (range)	0.35 (0.22–0.75)	0.29 (0.2–0.75)	0.51 (0.27–0.82)	0.09
hsTnl, pg/mL (range)	9.5 (5.1–32.9)	7.9 (4.9–15.4)	22.6 (10.7–114)	0.001
NT-proBNP, pg/mL (range)	202 (87–1145)	175 (63–605)	737 (220–2103)	0.001
Creatine kinase, U/L (range)	108 (47.6–196.4)	82 (44.4–194)	141.5 (80.4–217.7)	0.12
CK-MB fraction, U/L (range)	1.59 (0.83–4.7)	1.5 (0.7–4.8)	2.1 (1.1–3.6)	0.77
Ferritin, ng/mL (range)	481.7 (249–894.4)	457 (196.3–840)	629.7 (320.9–1290)	0.02
Fibrinogen, g/L (range)	4.96 (3.87–6)	4.6 (3.7–5.5)	5.74 (5–7)	0.001
Alkaline phosphatase, U/L	81.5 (65–113)	79.7 (61.9–102.8)	97.35 (76.1–125.5)	0.02
LDH, U/L (range)	310 (222–440)	274 (203.7–371)	394 (264–473.1)	0.001
AST, U/L (range)	37.6 (20.9–49.9)	35.8 (21.7–54.7)	37.6 (25–56.8)	0.64
ALT, U/L (range)	31.5 (21–49.9)	33.2 (20.9–53.9)	28.9 (21.6–44.8)	0.37
Direct bilirubin, mg/dL	0.16 (0.11–0.23)	0.14 (0.11–0.22)	0.19 (0.11–0.26)	0.12
Total bilirubin, mg/dL	0.66 (0.47–0.97)	0.67 (0.47–0.93)	0.65 (0.47–1.05)	0.89
Albumin, g/L (range)	3.53 ± 0.59 *	3.68 ± 0.57 *	3.11 ± 0.41 *	0.56 ^†^
INR (range)	1.11 (1.02–1.2)	1.1 (1–1.2)	1.16 (1.06–1.23)	0.12

Values are expressed in number (percentage) and median (interquartile range). AST = aspartate aminotransferase, ALT = alanine aminotransferase, CK-MB= creatine kinase-MB, eGFR = estimated glomerular filtration rate, FiO_2_ = fraction of inspired oxygen, hsTnI = high-sensitivity Troponin-I, INR = international normalized ratio, LDH = lactate dehydrogenase, NT-proBNP = NT-pro-brain natriuretic peptide, PaO_2_ = partial pressure of oxygen, * media (Standard deviation), ^†^ Student’s *t*-test, ^§^ Mann–Whitney U test.

**Table 3:** Indexes on admission in patients with COVID-19.

Variable	Total (*n* = 109)	Critical Illness		*p*	Death Total 19 (17)
		No (*n* = 77)	Yes (*n* = 32)		
**CURB 65**					
0	39	34 (44)	5 (16)	0.009	1 (2.5)
1	35	30 (39)	5 (16)	0.02	5 (14)
2	29	11 (14)	18 (56)	0.0001	9 (31)
3	3	1 (1)	2 (6)	0.20	2 (67)
4	3	1 (1)	2 (6)	0.20	2 (67)
**COVID-GRAM**					
Riesgo bajo <1.7%	4 (4)	4 (5)	0	0.3	0
Riesgo intermedio (1.7–40.4%)	75	60 (78)	15 (47)	0.002	10 (13)
Riesgo alto >40.4%	30	13 (17)	17 (53)	0.0003	9 (30)
**NEWS 2 score**					
Low risk (0–4)	37 (34)	36 (47)	1 (3)	0.0001	1 (3)
Moderate risk (5–6)	19 (17)	18 (23)	1 (3)	0.01	0
High risk (>7)	52 (48)	22 (29)	30 (94)	0.0001	18 (35)
**Berlin criteria**					
Without risk	24 (21)	24 (31)	0	0.0001	0
Mild	3 (3)	2 (3)	1 (3)	NS	0
Moderate:	61 (56)	47 (61)	14 (44)	NS	8
Severe:	21 (19)	4 (5)	17 (53)	0.0001	11
**Rox index**					
Minor risk for intubation	74 (68)	77	30	0.08	1
High risk for intubation	33 (30)	0	2	0.08	1
**Padua Score**					
≤4 points low risk	11	10	1	NS	0
≥4 points high risk	98	67	31	NS	19
**Q (CSI) index**					
≤3 Low (4%)	23 (21)	23 (30)	0	0.0002	0
4–6 Low-intermediate (30%)	22 (20)	17 (22)	5 (16)	0.6	3 (14)
7–9 High-intermediate (44%)	16 (15)	14 (18)	2 (6)	0.14	1 (6)
10–12 High (57%)	48 (44)	23 (30)	25 (78)	0.0001	15 (94)
Score C4					
Low	17 (16)	17 (22)	0	0.0002	0
Intermedium risk	36 (33)	31 (40)	5 (16)	0.01	3 (8)
High	48 (44)	27 (35)	21 (66)	0.005	12 (25)
Very High	12 (7)	2 (3)	6 (19	0.007	4 (33)

Mild: PaO₂/FiO₂ > 200 to ≤300 mmHg with PEEP OR CPAP ≥ 5 cm H₂O, Moderate: PaO₂/FiO₂ > 100 to ≤200 mmHg with PEEP ≥ 5 cm H₂O, Severe: PaO₂/FiO₂ ≤ 100 mmHg with PEEP ≥ 5 cm H₂O, Rox index ≥4.88 minor risk for intubation, ≤3.85 High risk for intubation, Padua Score ≥ 4points—pharmacologic prophylaxis is indicated. If high risk of bleeding, use mechanical prophylaxis. Padua Score < 4 points—pharmacologic prophylaxis is not indicated; consider using mechanical prophylaxis. The Quick COVID-19 Severity Index (qCSI).

**Table 4:** Chest-computed tomography findings on admission in patients with COVID-19.

		Critical Illness		
Chest CT Findings, No. (%)	Total (*n* = 109)	No (*n* = 77)	Yes (*n* = 32)	*p*
GGO	98 (89.9)	67 (87)	31 (96.8)	0.10 ^¶^
Consolidation	61 (55.9)	36 (46.7)	25 (78.12)	0.001 ^‡^
Crazy paving pattern	6 (7.5)	3 (5.4)	3 (12)	0.27 ^¶^
Linear pattern	41 (37.6)	27 (35)	14 (43.7)	0.39 ^‡^
Lymphadenopathy	32 (29.36)	19 (24.6)	13 (40.62)	0.09
Pleural effusion	22 (20.18)	7 (9)	15 (46.8)	0.001
Pericardial effusion	17 (15.6)	5 (6.49)	12 (37.5)	0.001 ^¶^
Pulmonary fibrosis	3 (2.78)	1 (1.32)	2 (6.25)	0.20
Pneumothorax	1 (0.92)	1 (1.3)	0	0.70
Steatosis	23 (21.3)	18 (23.6)	5 (15.62)	0.25 ^‡^
Emphysema	2 (1.83)	0	2 (6.25)	0.08 ^¶^
**CO-RADS, No. (%)**				
CO-RADS 0	0	0	0	0
CO-RADS 1	7 (6.42)	6 (7.79)	1 (3.12)	0.33
CO-RADS 2	0	0	0	
CO-RADS 3	7 (6.42)	5 (6.49)	2 (6.25)	0.66
CO-RADS 4	18 (16.51)	14 (18.18)	4 (12.50)	0.46 ^‡^
CO-RADS 5	54 (49.5)	36 (46.75)	18 (56.25)	0.36
CO-RADS 6	23 (21.10)	16 (20.78)	7 (21.88)	0.89
**Distribution, No. (%)**				
Peripheral distribution	55 (50.46)	43 (55.84)	12 (37.50)	0.08
Central distribution	22 (20.18)	11 (14.29)	11 (34.38)	0.01
Peripheral and central distribution	20 (18.35)	12 (15.58)	8 (25)	0.24
None	12 (11.01)	11 (14.29)	1 (3.12)	0.08 ^¶^
**CT score, Mean (SD)**				
Left upper lobe	2.33 (1.48)	1.96 (1.29)	3.25 (1.54)	0.001 ^†^
Left lower lobe	3.12 (1.69)	2.78 (1.61)	3.93 (1.61)	0.00
Right upper lobe	2.36 (1.52)	1.97 (1.33)	3.32 (1.55)	0.00
Middle lobe	2.33 (1.55)	1.90 (1.37)	3.38 (1.47)	0.00
Right lower lobe	3.03 (1.57)	2.68 (1.56)	3.90 (1.22)	0.00
Total, severity score	14 (8–19) *	11 (7–16) *	20 (16–23) *	0.0001 ^§^

Values are expressed in number (percentage) and mean (standard deviation). CO-RADS = COVID-19 reporting and data system, CT = computed tomography, GGO = ground glass opacity, IQR = interquartile range. * median (IQR), ^†^ Student’s *t*-test, ^‡^ chi-squared test, ^§^ Mann–Whitney U test, ^¶^ Fisher’s exact test.

**Table 5:** Risk factors for critical illness.

	Univariate Analysis		Multivariate Analysis	
**Variables**	**OR (95 % CI)**	***p***	**OR (95 % CI)**	***p***
Age, years	1.03	0.02		0.14
**Laboratories findings**				
PaO_2_, mmHg	0.96	0.00	0.96	0.05
FiO_2_, %	1.11	0.00	1.11	0.001
Neutrophil cell count, x10^3^/L	1.23	0.00		0.33
Neutrophil–lymphocyte ratio	2.19	0.00	1.94	0.001
Potassium, mmol/L	2.02	0.01		0.86
Calcium, mg/dL	0.43	0.01		0.53
eGFR, ml/min/1.73 m^2^	0.98	0.01		0.17
Fibrinogen, g/L	1.10	0.01		0.18
**Chest CT findings**				
Consolidation	4.06	0.00		0.11
Pleural effusion	8.82	0.00		0.06
CT score ≥18	13.82	0.00	5.13	0.01
**Risk clinical Scores**				
High risk COVID-GRAM	5.57	0.00		0.07
High risk qCSI	5.83	0.00		0.07

CI = confidence interval, COVID-GRAM = critical illness risk score, eGFR = estimated glomerular filtration rate, FiO_2_= fraction of inspired oxygen, OR = odds ratio, PaO_2_ = partial pressure of oxygen, qCSI = quick COVID-19.

**Table 6:** Performance characteristics of COVID-19 severity risk scores and total severity scores by CT in the prediction of critical illness.

	AU-ROC	Sensitivity	Specificity	Positive LR	Negative LR	OR	PPV	NPV
NLR severe	0.64	34.4	94.8	6.62	0.69	9.56	73.9	77.7
CT score ≥ 18	0.78	71.9	84.4	4.61	0.33	13.8	66.4	87.5
High risk COVID-GRAM	0.68	53.1	83.1	3.15	0.56	5.58	57.4	80.5
High risk qCSI	0.68	84.4	51.9	1.76	0.30	5.84	42.9	88.6
Combination of clinical scores and TSS *								
NLR severe plus TSS	0.77	57.1	98.4	36	0.43	82.7	93.9	84.3
High risk COVIDGRAM plus CT score ≥ 18	0.83	72.2	94.8	14	0.29	47.7	85.7	88.8
High risk qCSI plus CT Score total	0.85	90.9	80.4	4.65	0.11	41.1	66.6	95.4

* total severity score greater than 18 AU-ROC = area under the ROC, CT = computed tomography, COVID-GRAM = critical illness risk score, LR = likelihood ratio, NLR = neutrophil–lymphocyte ratio, NPV = negative predictive value, OR = odds ratio, PPV = positive predictive value, qCSI = quick COVID-19 severity index.

## Data Availability

The data in our study are available from the corresponding author upon reasonable request. MES.
